# BitterBuster: A virtual world for understanding naturalistic patch foraging

**DOI:** 10.1371/journal.pone.0361502

**Published:** 2026-10-30

**Authors:** John P. Kasarda, Jyotika Bahuguna, Angela Zhang, Hua Tong, Yuan Tan, Ruizi Wang, Michael J. Tarr, Timothy Verstynen

**Affiliations:** 1 Department of Psychology, Carnegie Mellon University, Pittsburgh, Pennsylvania, United States of America; 2 Laboratoire de Neurosciences Cognitives et Adaptatives (LNCA), Faculté de Psychologie, Université de Strasbourg, Strasbourg, France; 3 Entertainment Technology Center, Carnegie Mellon University, Pittsburgh, Pennsylvania, United States of America; 4 Neuroscience Institute, Carnegie Mellon University, Pittsburgh, Pennsylvania, United States of America; MARE – Marine and Environmental Sciences Centre, PORTUGAL

## Abstract

The past decade has seen a resurgence of interest in the naturalistic behavior of patch foraging in both the neural and cognitive sciences. Patch foraging is observed throughout the animal kingdom and requires the integration of multiple cognitive processes, including value and perceptual learning, spatial estimation, the evaluation of instantaneous return rates, and anticipating future effort. Here, we present a novel virtual environment, BitterBuster, for studying patch foraging in humans. In the BitterBuster game, players navigate a virtual world with four neighborhoods (patches) populated with houses (harvest points) containing inhabitants known as “Oomplets”. The goal is to search for “bitter” Oomplets, characterized by the union of specific body colors and textures. Each neighborhood has a different overall probability of containing bitter Oomplets and, within each, specific house locations predict the relative rate of bitter Oomplets. BitterBuster can be used for solo or team based foraging, where the tasks of exploring and perceptual learning are implemented by two separate players working together. Our results show how both the solo and team version of BitterBuster can accurately measure multiple aspects of naturalistic patch foraging, with participants learning to identify optimal patches, classify harvestable resources, and make rational decisions about when to leave a patch before overharvesting. BitterBuster is well-suited for researchers interested in the contextual, neural, and cooperative dynamics of foraging.

## Introduction

Consider a lemur deciding which tree to climb in order to consume a fruity lunch. She must choose which trees are likely to have plenty of ripe fruit that is easily accessible. Once she picks a target and climbs it, she must learn both the structure indicating where fruit is likely to be found on the tree and the perceptual cues for identifying which fruit is ripe enough to eat. She must also decide at what point she has eaten most of the available fruit on a given tree and whether it is time to move on to a potentially more plentiful tree. This nested set of interacting choices reflects the challenge of patch foraging [1].

Nearly all mobile animals engage in some form of patch foraging [2–4]. Nematodes search for patches of bacteria prey, mice explore restaurants for scattered patches of food scraps, and humans spend hours moving from one Wikipedia article to the next. Patch foraging differs from typical decision science problems because it involves cyclically dependent choices: accept or reject a resource patch (*patch selection*) and, if accepted, decide how long to exploit it before moving on (*patch exit*) [1,5]. This basic structure provides a foundation for ecologically relevant choices and differs substantially from the typical n-choice paradigms used to investigate decision-making in the lab [6].

The vast majority of experimental work has focused on individual components of the foraging process in isolation. For example, ecological and neurocomputational accounts have formalized patch residence time and switching in terms of marginal returns, opportunity costs, and reinforcement learning [5,7–9]. In contrast, as with our foraging lemur, naturalistic harvesting also depends on multiple forms of learning: the forager must acquire perceptual expertise about resource identity [10] (e.g., ripe or not), and often must discover latent spatial or structural regularities that make some locations within a patch more profitable than others [11] (e.g., which kinds of branches are likely to hold fruit). Experimental platforms that jointly manipulate these components while keeping them analytically separable remain relatively rare, particularly when locomotor exploration and perceptual classification occur in separate visual workspaces [12].

Patch foraging can also be formulated as a team problem. Social foragers, like stickleback fish, use public information from conspecifics to infer patch quality [13,14]. Group settings often distribute search and exploitation across complementary roles, as captured by producer-scrounger dynamics [15]. In human foraging teams, and increasingly in hybrid human-AI teams, the relevant information may be partitioned across team members rather than contained in a single individual: one teammate may be better at sampling the environment, whereas a second teammate may be better at classifying resources or estimating resource uncertainty [16,17]. Given these constraints, an effective virtual environment for studying patch foraging should support both individual and role-specialized team variants of the same task.

To this end, we present BitterBuster as an easy-to-use resource for studying naturalistic patch foraging in a controlled, but intuitive, virtual environment. Participants navigate a candy-themed world with four neighborhoods (patches) containing houses (harvest points) that vary in both overall and local reward probabilities, and harvest serially presented “Oomplets,” whose bitter/sweet category must be learned from perceptual features [18]. BitterBuster offers both a solo version, in which one participant performs exploration and harvesting, and a team version, in which exploration and selection are split across two participants. Across both versions, BitterBuster allows experimenters to manipulate patch-level value, within-patch structure, travel costs, time pressure, and information asymmetries while retaining common behavioral measures.

The experiments presented here serve as an initial validation of the BitterBuster platform. We use the current data to both adjudicate among theories of foraging and to show that BitterBuster elicits canonical patch-foraging behavior that can be dissociated into multiple learning processes relevant to foraging performance. This positions BitterBuster as a reusable task for future work on solo foraging, social coordination, and potential human-AI teaming.

## Results

### Experiment 1: Solo foraging

Within the solo version of BitterBuster, participants were instructed to find and correctly identify as many “bitter Oomplets” as possible within the time allotted for each round. Bitter Oomplets were defined by a conjunction of visual features that participants had to learn from trial-and-error. Participants explored four neighborhoods within a virtual candy world to find and correctly classify Oomplet targets (Fig 1A). Starting in a central arena, participants selected and moved into a neighborhood (patch selection) and then approached one of the houses scattered around that neighborhood (colored points in the inset of Fig 1A). A house could be explored by entering it, at which point the participant could harvest Oomplets. For this, a new window was displayed in which participants were presented serially with images of Oomplets that were bitter (Fig 1B) or sweet (Fig 1C). Participants made a binary classification based on these perceptual features and were given feedback on whether their choice was correct.

**Fig 1:**
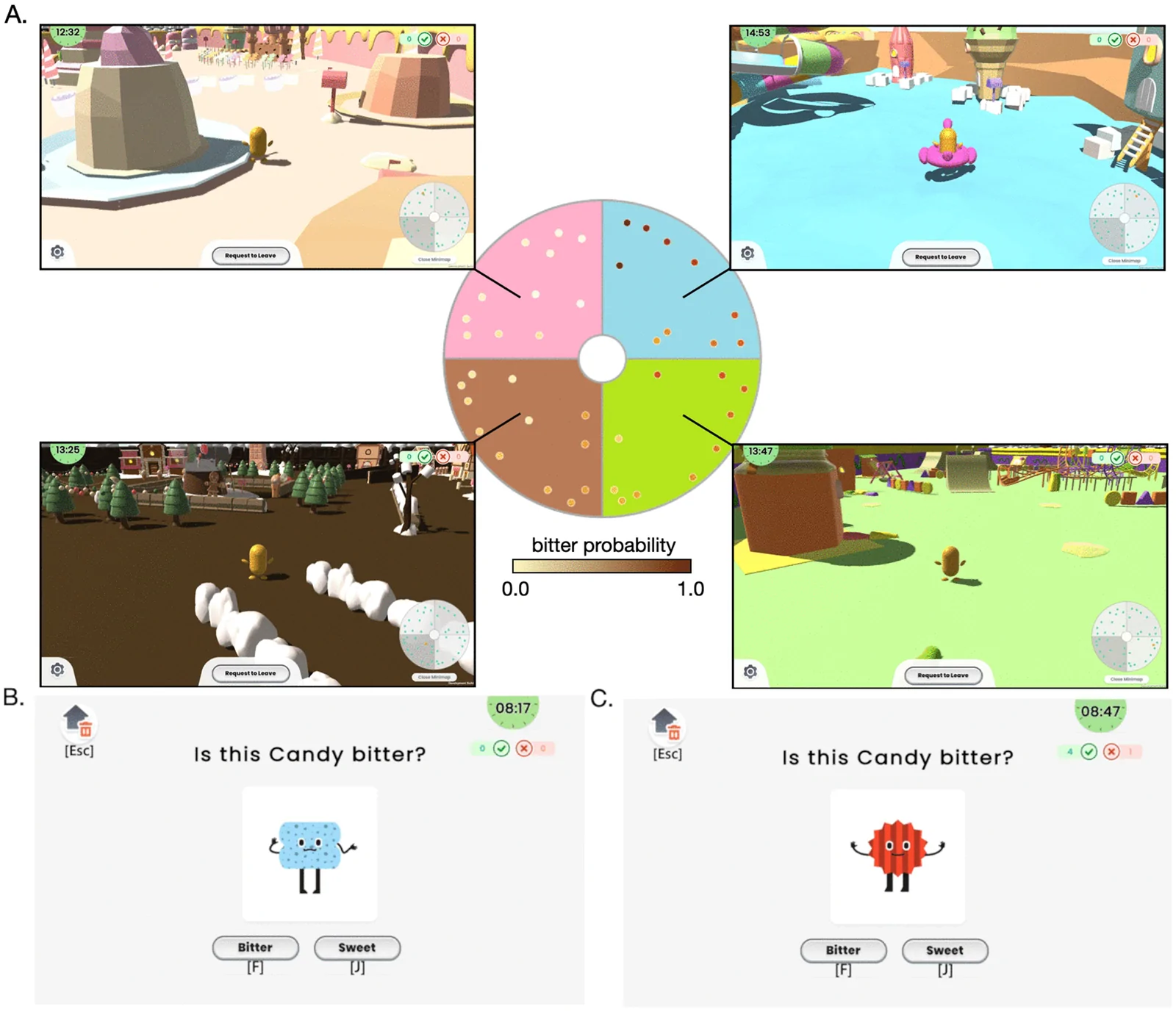
BitterBuster player interface. (A) Bird’s-eye view of the Oomplet kingdom, and screenshots of the four neighborhoods. Each neighborhood is a resource patch to be harvested. Within each patch are harvest points that can be explored, in the form of houses containing harvestable Oomplets. (B) A bitter Oomplet, here defined by cooler colors with spotted textures, in the selection task window. (C) A sweet Oomplet, here defined as any other combination of features, in the selection task window.

Participants continued classifying Oomplets until all Oomplets inside a given house had been categorized or they chose to exit that house. The number of Oomplets per house – which was not disclosed to participants – ranged from 8–12. In general, most participants completed the classification task at each house, with only 15% of participants abandoning a house before classifying every Oomplet, and only 3% of participants doing so more than once. After exiting a house, the participant’s view returned to the neighborhood screen, where participants could either choose to continue to different house or exit the neighborhood. A selection round ended when either all available Oomplets had been harvested across all neighborhoods or the time limit was reached. At the end of each round, participants received feedback indicating the proportion of bitter Oomplets they correctly identified (hits) and the proportion of sweet Oomplets incorrectly identified as bitter (false alarms). At the start of each round all houses were repopulated with Oomplets using the same sampling probability for all testing rounds. Participants (N = 60) completed three rounds of varying duration (20 minutes, 10 minutes, 10 minutes). Durations were communicated to participants prior to testing. The first round was longer to allow sufficient time for more thorough exploration of the BitterBuster environment.

In our analyses each visit to a house was treated as a trial and each *correctly* classified bitter Oomplet was treated as a reward. Examining average rewards collected as a function of time, relative to resource patch (neighborhood) abandonment, reveals two patterns (Fig 2A). First, rewards increased with experience across Rounds 1–3 independent of when participants chose to leave a patch. Second, rewards show a small dip immediately prior to patch abandonment, reflecting a decline in harvesting rate as participants progress through a patch.

**Fig 2:**
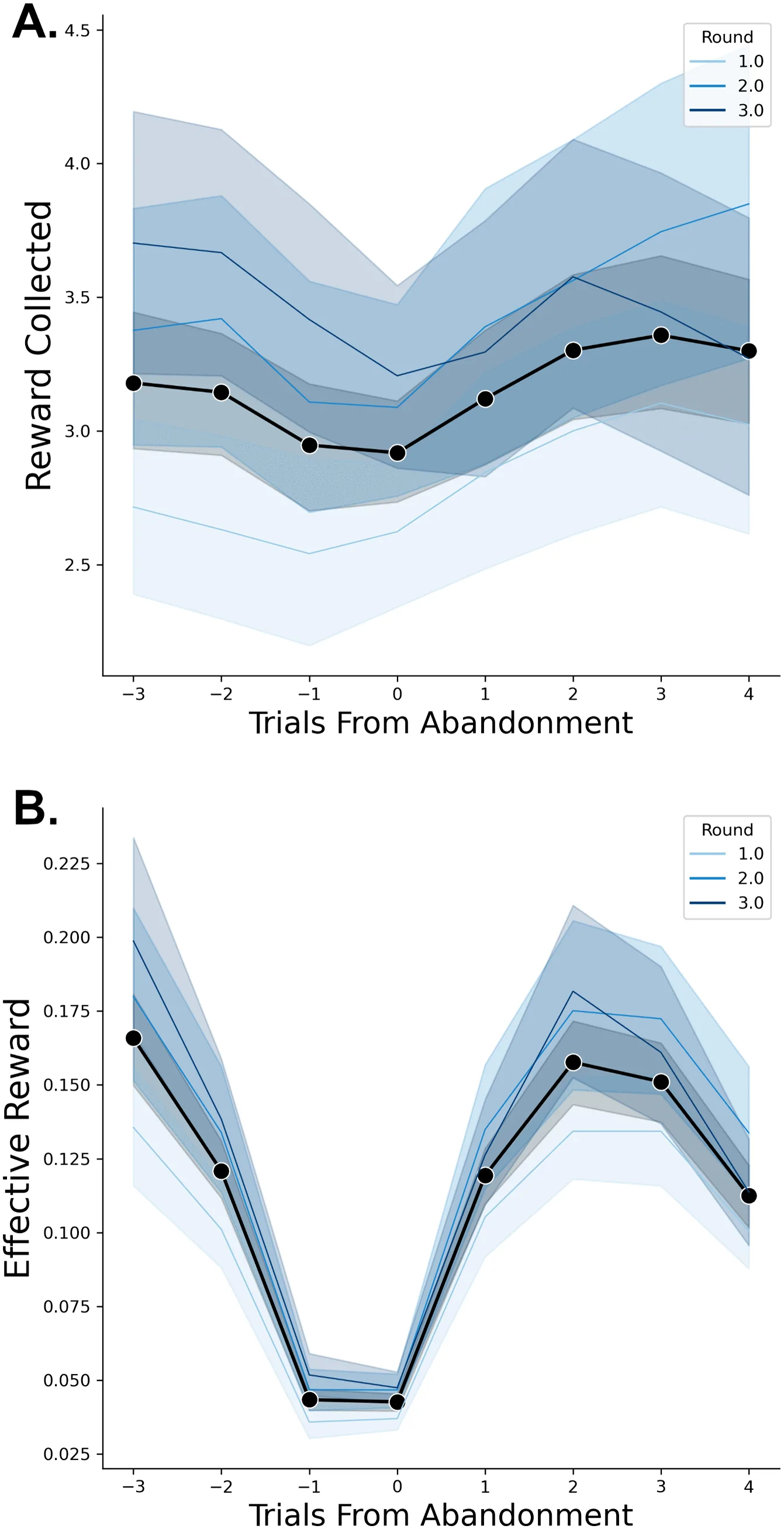
Experiment 1. Effective foraging rate. Foraging performance as a function of time relative to resource patch (neighborhood) abandonment. Color saturation indicates Round number, whereas the black line indicates average performance across all rounds. (A) Collected rewards – correctly classified bitter Oomplets – per house visit. (B) Expected reward rate – the rate of correctly classified bitter Oomplets – as a function of the distance between houses.

The Marginal Value Theorem [5] (MVT) states that optimal patch foraging requires: 1) estimating the relative effort for moving patches; 2) a balance of overall reward rate against the immediate returns gained at a given patch. The first prediction of the MVT is that there is a trade-off associated with estimating future effort of transitioning patches. To capture this tradeoff we define a measure of “effective reward rate” (see *Methods*). This rate reflects the immediate return on rewards (i.e., correctly identified bitter Oomplets), normalized by the distance between houses, approximating the effort to travel to a house with a new set of Oomplets. One prediction from the MVT is that, as participants harvest more houses within a neighborhood, the effective reward rate should decline as the distance to available houses with Oomplets increases since these are located in other neighborhoods. As such, effective reward rate should drop the closer a participant gets to abandoning a neighborhood (patch) and then increase once they move to a new neighborhood. Indeed, this is precisely what we see (Fig 2B). The drop in the effective reward rate near patch abandonment was significantly higher than the drop in the overall collected reward, indicating that participants were tracking the effort needed to get to available houses with Oomplets in the other neighborhoods when making their harvesting decisions.

Comparing the effective reward rate at the trial preceding a neighborhood abandonment versus the average reward collected on that trial, we observe a non-monotonic relationship that saturates at higher rates of effective rewards (Fig 3A-3C). Moreover, this relationship strengthens with experience from Rounds 1–3. This pattern indicates that harvesting becomes less efficient at the end of a patch harvest. Prematurely exiting from a patch (effective rewards of 0.00−0.02) and lingering in a patch too long (effective reward > 0.08), both lead to lower collected rewards. This bottleneck on harvesting efficiency also gets stronger with experience, indicating that participants are learning the return rate for each patch.

**Fig 3:**
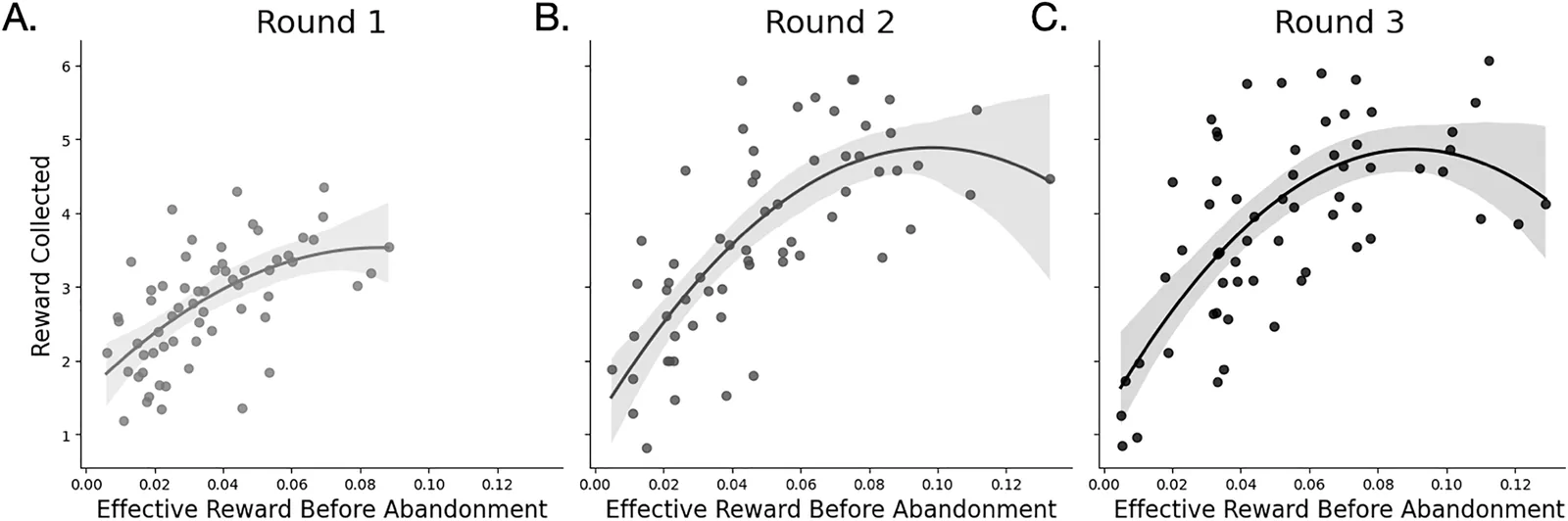
Experiment 1. Effective foraging rate. The relationship between average collected rewards and average effective reward rate for (A) Round 1, (B) Round 2, and (C) Round 3. Each point represents the total average reward collected per participant in a given round versus the average effective reward before patch abandonment.

The results shown in Fig 2 and Fig 3 indicate that the effective reward rate increases with experience. Quantifying this effect, we found that as training progressed, the average effective reward rate increased from a mean of 0.078 in Round 1 to 0.117 in Round 3 (Fig 4A). In a linear, mixed-effects regression model, this difference was reflected as a reliable main effect of Round (β=0.02,SE=0.003,p<0.001). Thus, with experience, participants were able to learn to forage with increasing effectiveness in the BitterBuster task.

**Fig 4:**
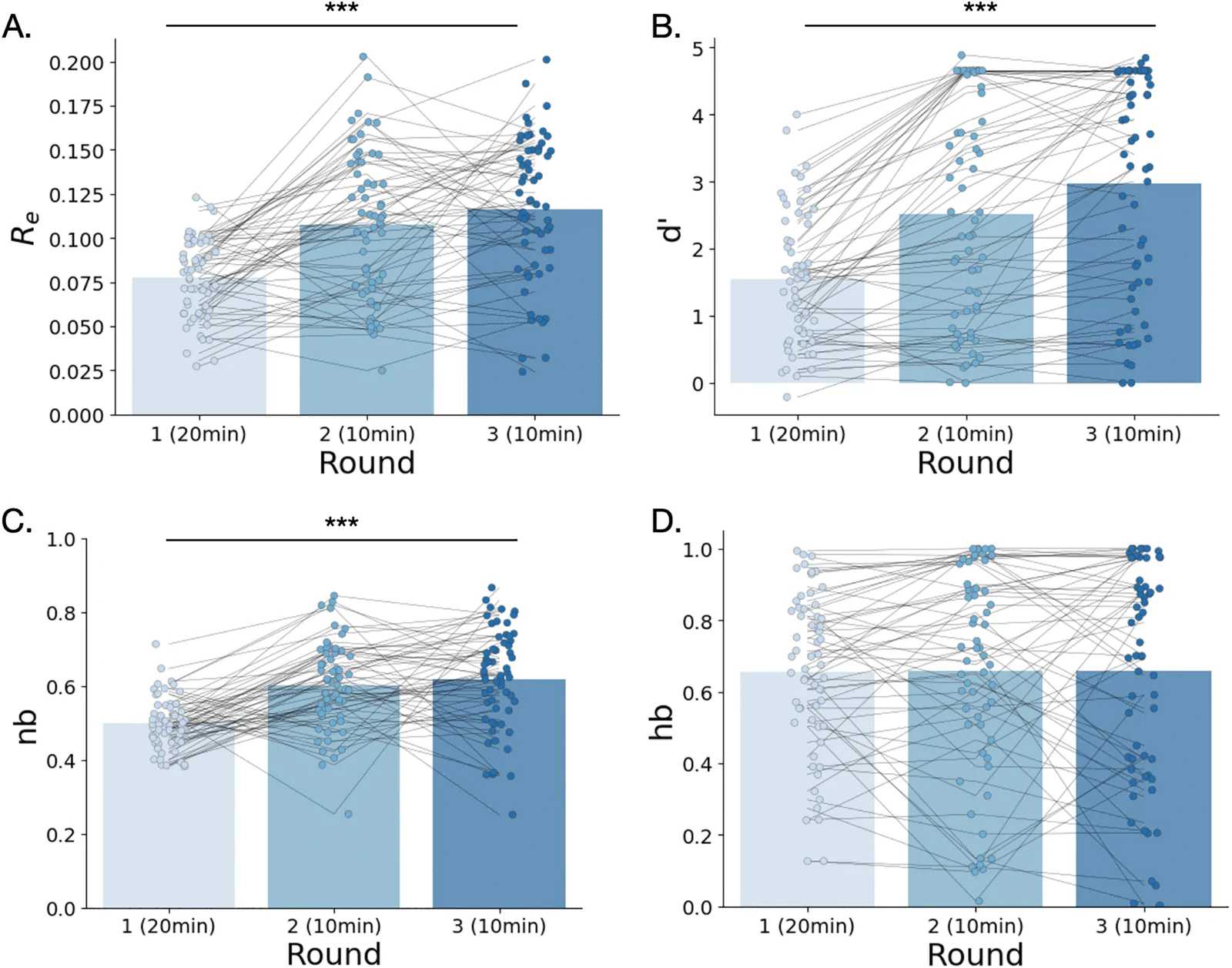
Experiment 1. Performance measure means and distributions. (A) Overall performance measured by Effective Reward; (B) Perceptual discrimination measured by $d^{\prime }$; (C) Patch value learning measured by Neighborhood Bias; (D) Patch structure learning measured by House Bias. The *** marker denotes a reliable difference, *p* < .001.

Next, we examined perceptual, value, and structure learning during the foraging task. To assess perceptual sensitivity over time, we calculated the $d^{\prime }$ for Oomplet classification (see *Methods*). Perhaps unsurprisingly, $d^{\prime }$ values increased with practice, beginning with an average of 1.55 in Round 1 and rising to 3.01 in Round 3 (β=0.72, *SE* = 0.07, *p* < 0.001 Fig 4B). The fact that their classification ability improved over time indicates that participants learned the perceptual features that distinguish bitter from sweet Oomplets (see also [18]).

To estimate value learning, we computed a neighborhood bias score reflecting the proportion of time spent in each neighborhood weighted by that neighborhood’s overall bitter Oomplet probability. This bias score ranges from 0 to 1, with higher values indicating that a participant spent more time in neighborhoods with higher probabilities of bitter Oomplets (see *Methods*). On average, neighborhood bias increased with experience, rising from 0.50 in Round 1 to 0.62 in Round 3 (β=0.23, *SE* = 0.01, *p* < 0.001 Fig 4C). Thus, participants were able to successfully learn to identify and value neighborhoods with higher likelihoods of having bitter Oomplets.

Finally, at a more granular level, we examined whether participants learned the within-neighborhood structural patterns that predicted bitter Oomplet probability. To assess this, we created a house bias score by ranking each house within a neighborhood according to its actual bitter probability and comparing that to the participant’s search order using a Spearman’s rank-order correlation coefficient to quantify their alignment (see *Methods*). In contrast to neighborhood bias, house bias did not show significant improvement with practice. The mean value was maintained at 0.66 through Rounds 1–3 (β=0.00, *SE* = 0.01, *p* = 0.684 Fig 4D). These results indicate that while participants were able to detect global patterns across neighborhoods, the finer-grained spatial patterns within neighborhoods were not reliably learned during the period of time allotted in our study (a single day of experience with the BitterBuster task).

To summarize our results thus far, we find that overall foraging efficiency in the BitterBuster task improves with practice. This improvement coincides with gains in perceptual discrimination and patch value learning, but not with learning of within-patch latent structure. While these results provide some insight into human patch foraging behavior, we were primarily interested in understanding how disparate learning abilities contribute to patch foraging effectiveness. To this end, we examined how effective reward rate relates to perceptual and value learning – the two cognitive abilities that exhibited practice-related improvements. Fig 5 shows the relationships between $d^{\prime }$, neighborhood bias, and effective reward rate for Rounds 1–3. These plots show the emergence of correlations between both $d^{\prime }$ and neighborhood bias with effective reward, from early in training (Round 1) to later in training (Round 3). These trends suggest that both perceptual and value learning may independently relate to foraging ability, but may also interact with one another.

**Fig 5:**
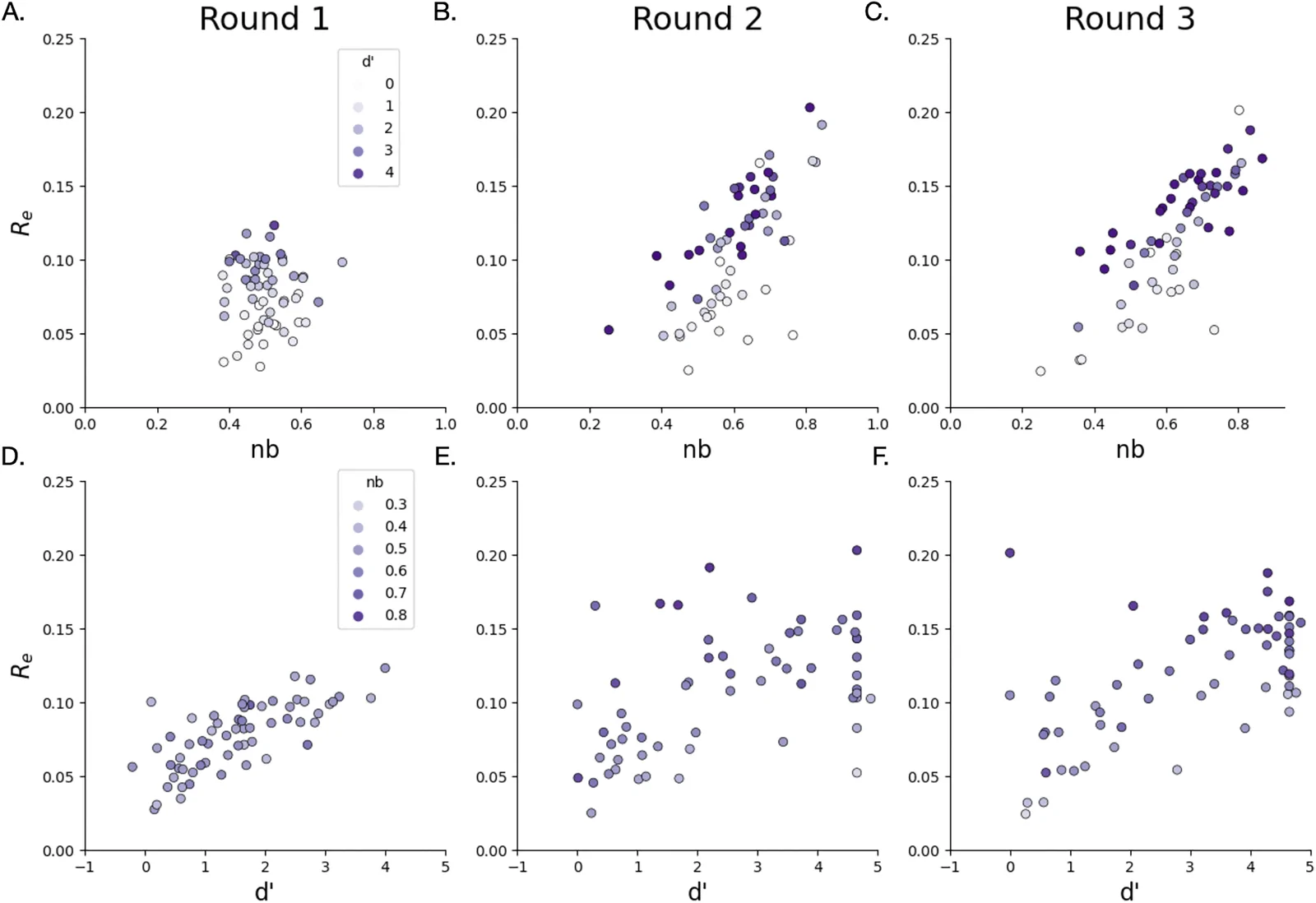
Experiment 1. Effective reward versus task performance measures for Rounds 1-3. (A-C). Exploration performance (neighborhood bias; *“nb”*) for Rounds 1-3. (D-F). Selection performance (*d’*) for Rounds 1-3.

To formally test this claim, we conducted a linear mixed-effects regression analysis to examine the influence of $d^{\prime }$ and neighborhood bias on effective reward (see *Methods*). There was a significant main effect of $d^{\prime }$ (β=0.01, *SE* = 0.00, *p* = 0.001) and a strong main effect of neighborhood bias (β=0.20, *SE* = 0.02, *p* < 0.001; Table 1). However, there was no significant interaction between these two factors (β=−0.003, *SE* = 0.00, *p* = 0.68). These results indicate that overall foraging effectiveness (i.e., effective reward) in the BitterBuster task most likely reflects the independent contributions of perceptual and value learning.

**Table 1:** Experiment 1. Mixed linear model regression for solo foraging.

	Coef.	Std.Err.	z	P > |z|	[0.025	0.975]
Intercept	−0.041	0.013	−3.260	0.001	−0.066	−0.016
Round	−0.001	0.002	−0.427	0.669	−0.005	0.003
d’	0.014	0.004	3.231	0.001	0.005	0.022
nb	0.200	0.023	8.667	0.000	0.155	0.246
d’:nb	−0.003	0.007	−0.419	0.675	−0.017	0.011
Group Var	0.000	0.004				

The second prediction of the MVT is that the patch exit decision should be driven by a comparison between the immediate returns on a patch and the overall expected return across all patches during the foraging session. The immediate gain dropping below the expected overall gain should trigger a decision to leave the current patch. As search pressures increase, foragers will typically leave patches before reaching this optimal point [19,20].

To capture this decision process, we define a measure of “marginal reward rate” (see Measures and Analysis). Once a participant enters the first house of a patch, a *patch reward rate* is estimated by the speed at which correctly identified bitter Oomplets are collected. This value is compared against a running tally of the overall rate of collecting bitter Oomplets across all patches during the session, called the *expected reward rate*. By subtracting the expected reward rate from the patch reward rate, we get the *marginal reward rate* which represents the instantaneous benefit or disadvantage to reward rate by staying on a given patch.

Fig 6A shows the estimated marginal reward rate time-locked to entering a patch. The gray histogram panels show the distributions of patch exit times for each round. Two patterns can be observed. First, as time constraints increase with each successive round, participants tend to exit patches earlier. This is reflected as a leftward shift of the distributions of exit times across rounds. Second, the marginal reward rate remains positive around exit times in the later rounds with tighter time constraints. During Round 1, the marginal reward rate settles near zero around the median exit time. For Rounds 2 and 3 these values remain positive, meaning that participants are leaving the patch even when the immediate reward rate is higher than the overall reward rate. This is also reflected as an increase in the marginal reward rates across the final thirty seconds before participants move into a new neighborhood (Table 2).

**Table 2:** Marginal reward rates at the end of a patch visit for solo foraging.

	Round 1	Round 2	Round 3
Mean	0.012	0.028	0.022
Median	−0.006	0.004	0.022
σ	0.078	0.100	0.101
SEM	0.005	0.007	0.007

**Fig 6:**
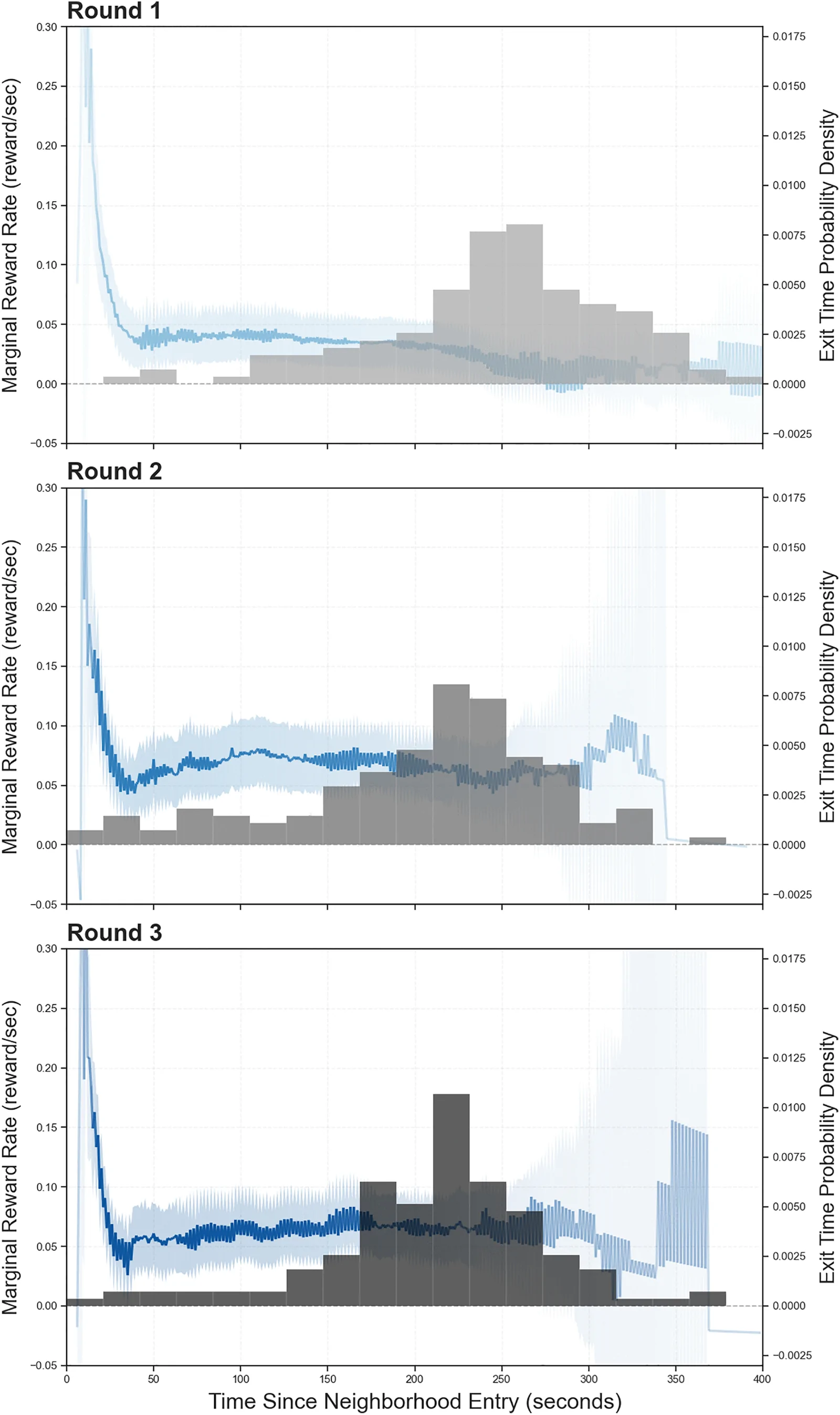
Experiment 1. Marginal reward rate for Rounds 1-3. Blue lines shows the immediate value of the marginal reward rate at each time point after a participant enters a patch. Error bars reflect the 95% confidence interval across all patch visits. The gray histogram represents the probability distribution of patch exit times.

Experiment 1 highlights how BitterBuster captures standard solo patch foraging behaviors. Participants appear to anticipate future travel costs when assessing the immediate value of a harvest point, and they can rapidly learn to identify more valuable patches. With weak time constraints, patch exit decisions appear to coincide with near-zero marginal reward rates – that is, the immediate reward rate on the patch matches or falls below the expected reward rate. However, as time constraints increase, participants leave patches early, forfeiting potential returns in order to move to new patches.

### Experiment 2: Team foraging

In Experiment 2, we used the teaming version of BitterBuster to separate out the exploration and selection roles. One participant (the Explorer) sought out Oomplets by navigating the virtual world, visiting neighborhoods, and selecting which houses to enter. The other participant (the Selector) would then receive a series of Oomplets to classify as bitter or sweet using the same concatenations of perceptual features as in Experiment 1. Importantly, the Selector observed the movements of the Explorer up until the point at which a set of Oomplets was sent to the Selector for categorization. The Explorer, on the other hand, had no view of the Selector’s screen, but could observe their performance via displayed feedback bars.

To allow communication between the Explorer and the Selector, we added two new elements to BitterBuster. First, the Explorer and Selector were required to agree to leave a neighborhood via a text prompt whenever one player wished to explore a different area. Second, as suggestions for where the Explorer should go, the Selector was able to place markers in the game world to indicate areas of interest. By introducing these communication methods, we hoped to obtain measures of trust and team dynamics between the two players. However, there was insufficient diversity in their responses to leave requests to ascertain any meaningful patterns: of the 231 leave requests, only 11 were rejected. Similarly, Selectors did not use the world marker system: only 19 Selectors ever placed a marker.

The team version of BitterBuster showed very similar performance patterns to the solo version (Experiment 1). Effective reward significantly increased with each round of testing (β=0.02, *SE* = 0.002, *p* < 0.001 Fig 7A), the Explorers learned to identify high value neighborhoods (β=0.06, *SE* = 0.02, *p* = 0.002 Fig 7C), and the Selector learned to accurately classify Oomplets using trial-and-error (β=0.82, *SE* = 0.06, *p* < 0.001 Fig 7B). Thus, when each role was assigned to separate participants, teams exhibited learning patterns similar to those of solo foragers, showing correlations between both $d^{\prime }$ and neighborhood bias with effective reward (Fig 8). As in Experiment 1, the teams did not learn to identify the relationship between house position and Oomplet probability within a neighborhood (not shown).

**Fig 7:**
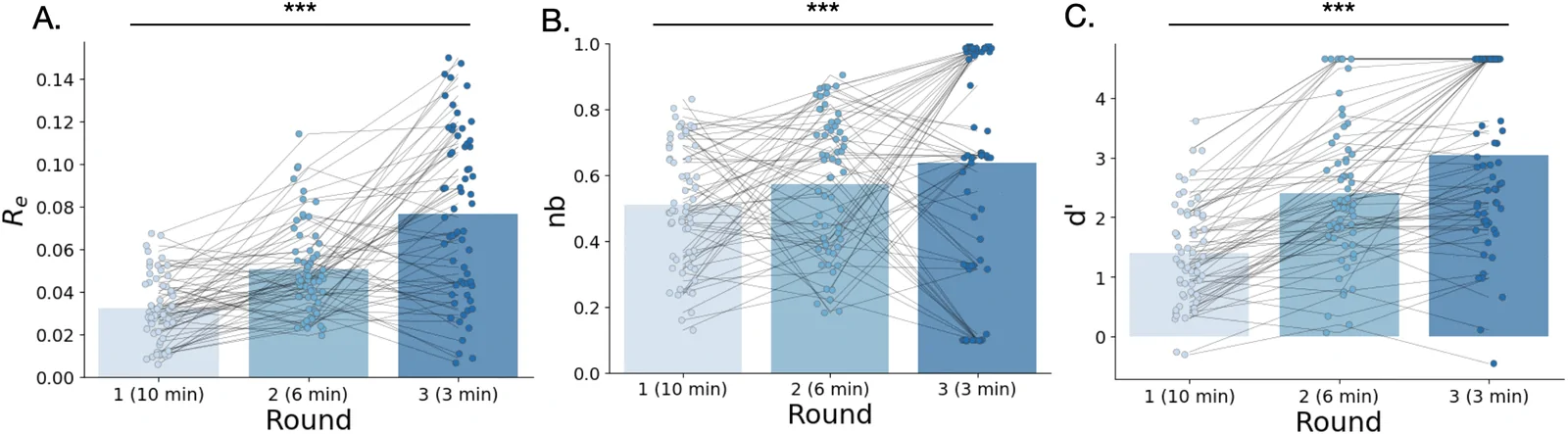
Experiment 2. Performance measure means and distributions for all teams. (A) Overall performance measured by Effective Reward; (B) Perceptual discrimination measured by $d^{\prime }$; (C) Patch value learning measured by Neighborhood Bias; (D) patch structure learning measured by House Bias. The *** marker denotes a reliable difference, *p* < .001.

**Fig 8:**
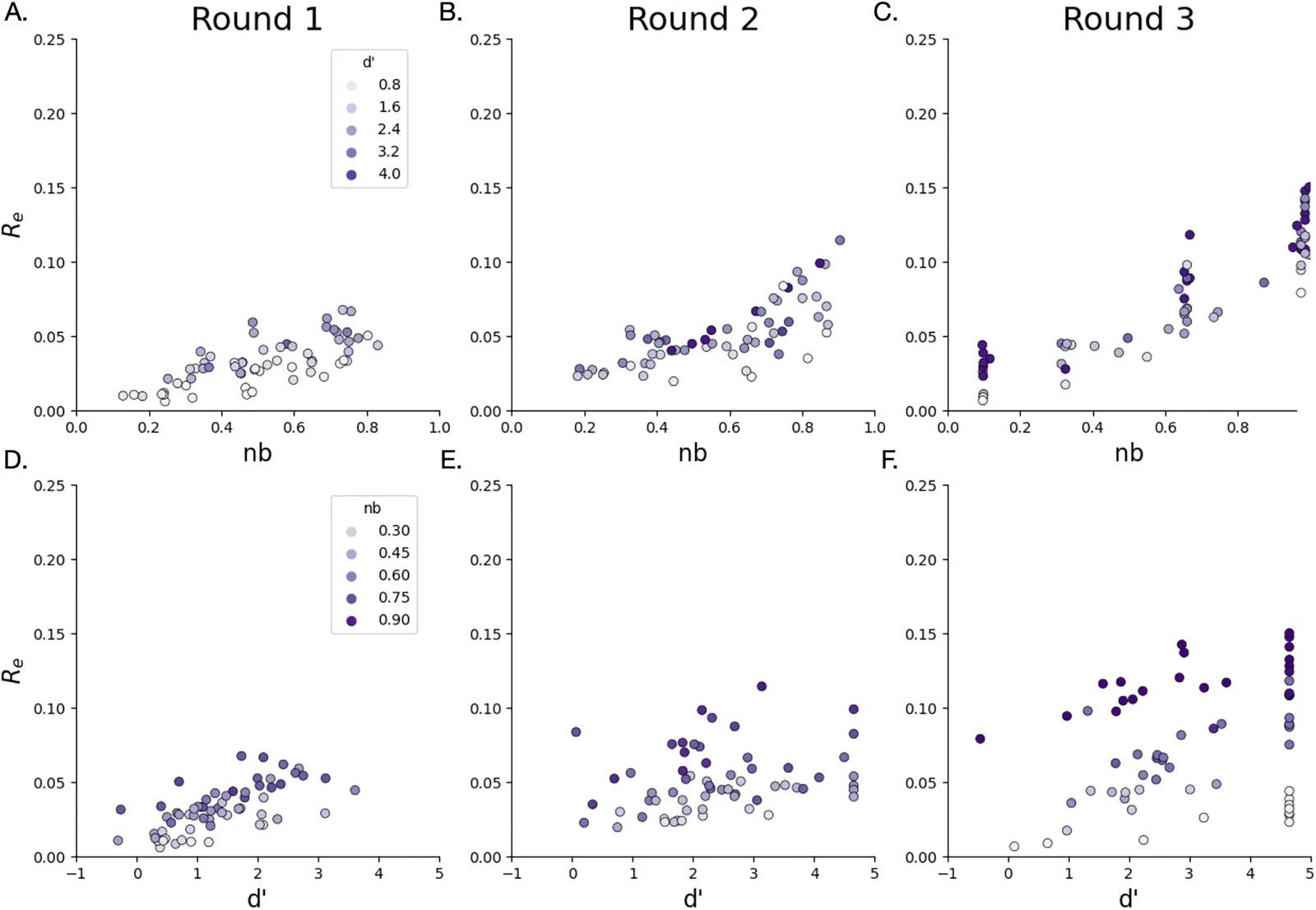
Experiment 2. Effective reward versus task performance measures for Rounds 1-3. (A-C). Exploration performance (neighborhood bias; *“nb”*) for Rounds 1-3. (D-F). Selection performance (*d’*) for Rounds 1-3. Color saturation represents the performance measure value from the complementary task.

In the teaming experiment, but not the solo experiment, we observed a significant interaction between the Explorer’s neighborhood bias score and the Selector’s perceptual classification performance ($d^{\prime }$) in predicting effective foraging (β=0.010, *SE* = 0.00, *p* < 0.001 Table 3). This positive interaction suggests that each teammate’s contribution to effective reward was moderated by the similarity in their skills. Specifically, stronger performance in both value learning and perceptual learning amplifies the team’s overall effective reward. Accordingly, the interaction term indexes the overall teaming effect in the BitterBuster task.

**Table 3:** Experiment 2. Mixed linear model regression for team foraging.

	Coef.	Std.Err.	z	P > |z|	[0.025	0.975]
Intercept	−0.021	0.005	−4.725	0.000	−0.030	−0.012
Round	0.012	0.001	8.835	0.000	0.009	0.014
d’	0.000	0.002	0.038	0.970	−0.003	0.003
nb	0.064	0.007	8.554	0.000	0.049	0.079
d’:nb	0.010	0.002	4.133	0.000	0.005	0.015
Group Var	0.000	0.001				

Patch exit times in the Experiment 2 teaming task qualitatively aligned with those observed in Experiment 1 (Fig 9). In particular, the timing of patch exits shifted earlier as time constraints increased. However, when the search window was narrow, as in Round 3, the round often finished before participants could exit the first visited patch. The marginal reward rate at the end of a patch visit again showed more positive values in later rounds with greater time pressures (Table 4), reflecting the foraging team leaving patches before being optimally harvested.

**Table 4:** Marginal reward rates at end of a patch visit for team foraging.

	Round 1	Round 2	Round 3
Mean	0.004	0.025	0.045
Median	0.003	0.027	0.047
σ	0.070	0.082	0.085
SEM	0.006	0.007	0.010

**Fig 9:**
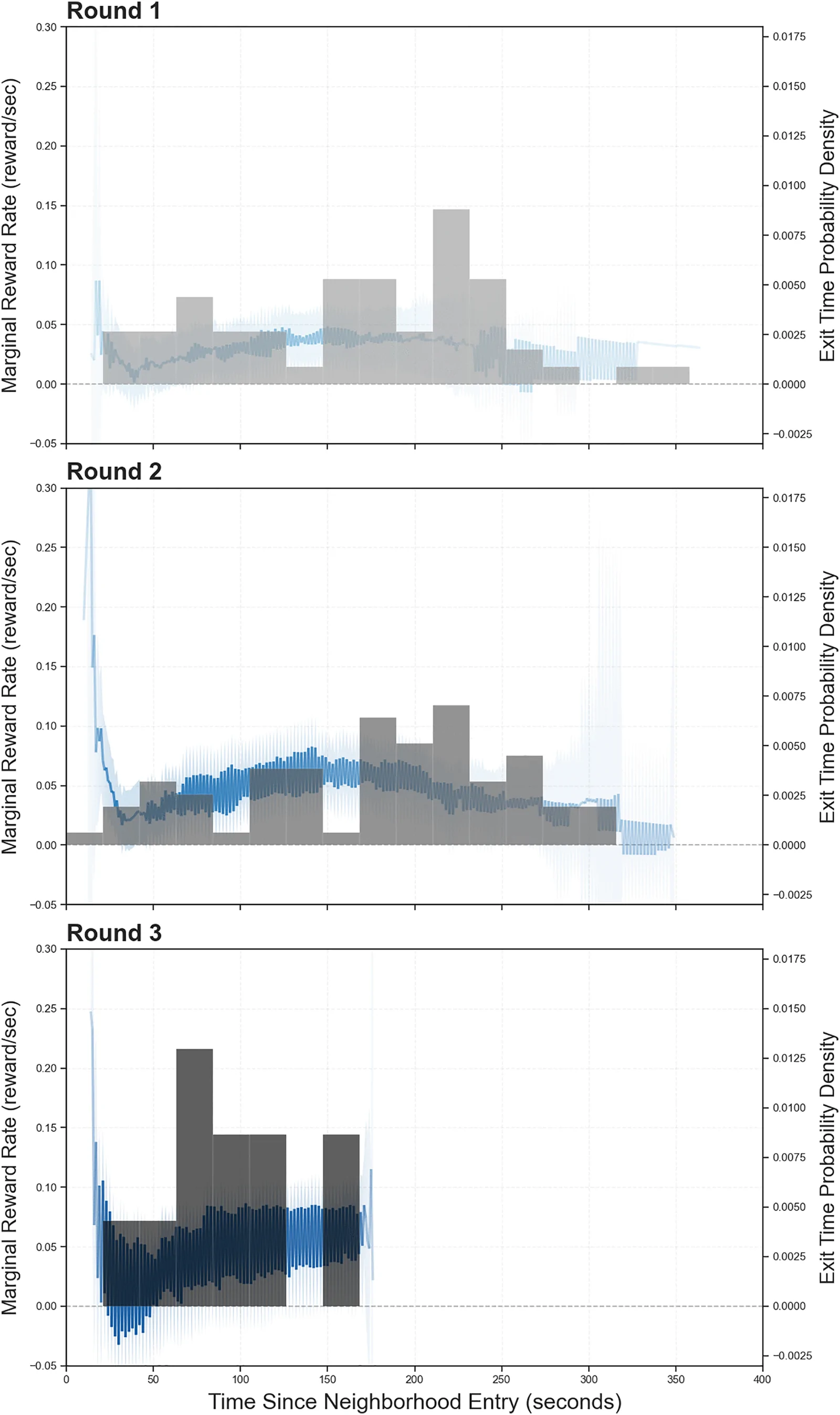
Experiment 2. Marginal reward rate for Rounds 1-3. Same plotting conventions as Fig 6.

Team foraging, as implemented in Experiment 2, revealed similar patterns to solo foraging. Teams took into account the effort to get to new patches and harvest points when estimating the value of harvesting decisions. Teams were able to estimate the relative value of different patches, but not the spatial structure within the patch. In contrast to solo foraging, teams showed an interaction between the performance of the Selector ($d^{\prime }$) and the performance of the Explorer (nb) on overall effective rewards. Finally, as time pressures increased, teams exhibited earlier exit times and positive marginal reward rates around the exit choices.

## Discussion

The primary contribution of this work is the introduction and initial validation of BitterBuster as a flexible platform for studying naturalistic patch foraging. The platform integrates features that are often separated across paradigms: movement-based search between patches, nested decisions within patches (neighborhoods) and harvest points (houses), trial-by-trial perceptual learning, controllable resource statistics, and a team-based implementation with asymmetric information. Our results demonstrate that these components are behaviorally tractable. Participants learned to distinguish bitter from sweet Oomplets, adjusted their behavior appropriately to the relative value of neighborhoods, and showed cost-sensitive changes in effective reward around patch abandonment. Together, these findings indicate that BitterBuster captures core properties of patch foraging as studied in the biological and decision sciences [2,5,20].

Our current experiments also provide useful calibration data for future researchers interested in using BitterBuster to understand naturalistic foraging behavior. Under the present timing and cue structure, the task was sensitive to perceptual and patch-level value learning, but a single session (round) was insufficient to produce reliable learning of the finer-grained house-level (harvest point) structure within neighborhoods. From a methodological point of view, this outcome suggests that the current parameterization of BitterBuster is well suited for studying how foragers integrate perceptual discrimination with patch choice, whereas studies focused on latent structure learning may benefit from longer training, more salient structural cues, greater depletion pressure, or explicit manipulations of volatility and memory load.

The Oomplet set size in each house is a task parameter that could be leveraged for studying nested, or hierarchical patch foraging [21]. Here we referred to houses as harvest points because participants did not appear to reliably treat them as resource patches in their own right. Only a small minority of participants ever left a house without classifying all Oomplets. However, this strategy might be modified by increasing the number of Oomplets at each house, so that participants feel pressure to leave before going through the entire set. Because these and other task dimensions are separably manipulable in BitterBuster, the platform can be tuned to target specific learning timescales within the broader patch-foraging problem.

The team version of BitterBuster expands its utility to social contexts. Social foraging research has shown that foragers can use public information from others to assess patch quality and that search and exploitation can become distributed across complementary roles [13–15]. BitterBuster provides a controlled framework for studying these questions by manipulating which participants can view the map, receive item-level feedback, request patch departure, and incur costs or delays in communication. This makes the task well-suited for investigating coordination, shared mental models, partner complementarity, and the conditions under which splitting exploration and selection roles across teammates improves or impairs overall performance.

A particularly promising direction for future experiments is human-AI teaming. Because exploration and selection are modularized in the task design, an artificial agent can be naturally inserted as either an Explorer or a Selector. An AI Explorer could navigate routes to neighborhoods and targets, as well as make leave/stay decisions, whereas an AI Selector could learn to classify Oomplets, communicate confidence, or flag uncertainty for human review. This creates a tractable framework for studying core questions in human-AI collaboration, including how people learn an AI teammate’s strengths and failure modes [16], whether maximizing standalone AI accuracy also maximizes team utility [17], when explanations improve coordination in *ad hoc* teams [22], and why subjective teammate preferences can diverge from objective team performance [23]. A shared patch-foraging environment with interpretable movement, choice, and reward metrics could therefore complement existing game-based human-AI benchmarks by incorporating ecological search, travel costs, and role asymmetries into the study of hybrid teams.

More broadly, BitterBuster provides a common experimental testbed for comparing formal accounts of foraging across individual and distributed settings. The same behavioral data can be analyzed using marginal value, reinforcement learning, Bayesian, or hybrid models, and the same world structure can be deployed across solo, human-human, and human-AI conditions. In this sense, our platform is designed not only to study foraging behavior within the task, but also to provide a controlled bridge between classical patch-foraging theory and emerging questions about distributed cognition and intelligent teammates. Our aim is that by releasing and framing the paradigm in this way, BitterBuster and its variants will be useful for researchers studying naturalistic foraging, collective decision-making, and adaptive human-AI systems.

## Methods

### Ethics statement

All experimental protocols were reviewed and approved by the Carnegie Mellon University Institutional Review Board (IRB). Written informed consent was obtained from all adult participants prior to participation in both single-player and team experimental sessions.

### Experiment 1: Solo foraging

#### Participants.

A total of 62 participants were recruited from the Carnegie Mellon University Psychology Department SONA System and compensated at a rate of 1 credit per hour. Data from two participants were excluded due to software misconfiguration or failure to perform the task, resulting in a final sample of 60 participants (36 female, 23 male, 1 declined to answer, mean age = 18.70 years). Recruitment began on September 24, 2024 (09/24/2024) and ended on October 23, 2024 (10/23/2024).

#### Task design.

Participants began each round in a small circular room at the center of a candy-themed world (“kingdom”) with four doors leading to separate neighborhoods. Each neighborhood contained nine to twelve houses inhabited by candy-like creatures called Oomplets – configurable humanoid characters designed for complex perceptual discrimination tasks [18]. The task was to locate and identify as many bitter Oomplets as possible based solely on perceptual features. Participants were not instructed on how to distinguish bitter from sweet Oomplets and instead relied on in-game feedback to guide learning (S1-S6 Figs includes all of the instruction slides shown to participants each round). Each of the four neighborhoods was associated with a different maximum probability of containing bitter Oomplets (1.0, 0.75, 0.50, and 0.25). Within neighborhoods, individual houses also varied in their probability of containing bitter Oomplets. The effective probability at each house was determined by the product of the neighborhood-level and house-level probabilities.

Within a neighborhood, the probability of bitter Oomplets at a house reduced in a clockwise direction relative to the entry point into that neighborhood (Fig 1A). The color of each house’s mailbox denoted the relative percentage of bitter Oomplets present in that house. Each mailbox used the same 3D model, but its color was changed along the blue-to-orange spectrum, corresponding to the portion of bitter Oomplets in that house. Bluer hues reflected higher bitter Oomplet probability such that a deep blue/purple mailbox indicated a subpatch with a majority of bitter Oomplets, a pink mailbox indicated an even distribution, and a light orange mailbox indicated a majority of sweet Oomplets. Participants were not explicitly informed about either the mailbox color or clockwise probability cues – thus, exploration was necessary to learn their significance.

The exploration of the candy kingdom was periodically interrupted by the selection task. This task began when participants entered a house by pressing the *e* key when they were in close proximity to the door. During this time, the screen would switch to a 2D display (Fig 1B and 1C) where Oomplets were presented one at a time and participants were asked to classify them by pressing the *f* key for bitter and *j* key for sweet. Immediately after each response participants were given visual and auditory feedback on the accuracy of their selection (auditory feedback was only given for correct responses); additionally, an accuracy counter was updated in the upper right of the screen. An Oomplet’s category was determined by the combination of two of ten possible attributes: color and pattern. Bitter Oomplets were those which had a bluish hue and a spotted background [18]; Oomplets with other colors and/or backgrounds were classified as sweet. A total of eight to twelve Oomplets were displayed sequentially for categorization before the participant was returned to the neighborhood.

Each session consisted of three rounds: 20 minutes for Round 1, 10 minutes for Round 2, and 10 minutes for Round 3. All rounds were otherwise identical. The extended duration of Round 1 allowed participants time to familiarize themselves with both the game’s keyboard controls and the game world layout. Rounds 2 and 3 were shortened to increase the urgency of search with the goal of revealing selection biases in behavior. If a participant visited all of the houses in every neighborhood before reaching the round time limit, the round was ended early; in total 29 participants in Round 1 and one participant in Rounds 2 and 3 visited every house, ending the respective round prior to reaching the time limit.

### Experiment 2: Team foraging

#### Participants.

A total of 120 participants were recruited from the Carnegie Mellon University Psychology Department SONA System and compensated at a rate of 1 credit per hour (N = 120, 79 female, 40 male, 1 declined to answer, mean age = 18.74 years). Recruitment began on September 12, 2023 (09/12/2023) and ended on October 11, 2023 (10/11/2023).

#### Task design.

To enable cooperative game play, we adapted BitterBuster’s design to accommodate a team of two participants. One participant, the *Explorer*, controlled the character, explored the virtual world, and decided which houses to investigate. Upon the Explorer entering a house, the Oomplet selection task was displayed to the second participant, the *Selector*. Until this point, the Selector observed the Explorer’s actions from a top-down view and was able to use their world marker to attempt to guide the Explorer. When the selection task was sent to the Selector, they were notified that the task was available, and were able to either begin the task or reject it. While the Selector was completing their task, the Explorer was able to continue exploring that neighborhood. The Explorer could queue up Oomplets from an additional house while the Selector was still categorizing from a previous house, but this queue was limited to one house at a time.

An additional mechanic in the Team foraging version was neighborhood locking: when the Explorer entered a neighborhood, a gate appeared behind them, preventing them from exiting to the central hub. Both participants had an interface button that said *Request to Leave*. Selecting this button would notify the participant’s teammate that they wished to leave the current neighborhood. If the teammate accepted the request, the Explorer would be transported to the central hub after a brief countdown. If the request was rejected, the Explorer would remain in their current position.

All other visual game assets remained unchanged between the solo and team foraging versions. The distribution probability of bitter and sweet Oomplets in the neighborhoods was the same for each participant pair in Experiment 2. Participants received only instructions relevant to their specific role and were naïve to their teammate’s task. Experiment 2 also used a different round duration pattern: 10 minutes for Round 1, 6 minutes for Round 2, and 3 minutes for Round 3. By progressively decreasing the duration of the rounds, we hoped to encourage optimization of role performance for both participants. The shorter overall durations also take into account the reduction in task complexity for the participants, as each teammate only had to learn one role.

### Measures and analysis

To measure the performance of the participants in the game, we calculated their *effective reward* rate, $R_{e}$, which measures the total amount of reward collected per unit of linear distance traveled by the participant.


$$\begin{array}{c} R_{e}=\frac{\sum R_{house}}{\sum \Delta d_{j-i}}=\frac{R_{T}}{D_{T}} \end{array}$$ (1)

Here $R_{t}$ is the running total of collected rewards (i.e., correctly identified Oomplets) up until the current time point in the round, and $D_{T}$ is the running total of distances between house visits, $\Delta d_{j-i}$, up to the current house. Effective reward captures performance improvements in both the exploration and selection tasks, and accounts for different time limits across the different rounds. In the case of the first patch visit, the center of the game world, where the player began each round, was used for the coordinates of the previous patch. In cases where the patches were located in separate neighborhoods, the players had to travel back to the center of the arena before entering a new neighborhood. As such, the distance between the houses in separate neighborhoods was calculated as a sum of distances from each house to the center point.

Perceptual learning was tracked using a signal detection measure, $d^{\prime }$, which is computed as the difference of the participant’s *z*-scores for hit and false alarm (FA) rates.


$$\begin{array}{c} d^{\prime }=\Phi^{-1}(hit)-\Phi^{-1}(FA) \end{array}$$ (2)

The hit rate was the proportion of total selection events in which a bitter Oomplet was shown and bitter was correctly selected. The false alarm (FA) rate was computed as the proportion of total selection events where a sweet Oomplet was shown and bitter was incorrectly selected. A $d^{\prime }>0$ indicates sensitivity in the intended direction, with increasingly stronger sensitivity as $d^{\prime }\to 1$; a $d^{\prime }\approx 0$ indicates no sensitivity; whereas a $d^{\prime }<0$ would indicate sensitivity opposite to the intended signal.

To measure how well participants learned the value of each neighborhood, we introduced a *neighborhood bias* (nb) score. This was calculated as:


$$\begin{array}{c} nb=\sum \frac{b_{n}t_{n}}{t_{T}} \end{array}$$ (3)

where $b_{n}$ is the bitter probability of neighborhood *n*, $t_{n}$ is the time spent in neighborhood *n*, and $t_{T}$ is the total round time. This weighs each neighborhood probability value against the time spent in the corresponding neighborhood and sums these weighted probabilities, resulting in a value between 0 and 1. *nb* was highest in rounds where a participant spent the majority of their exploring time in a high-value neighborhood.

To measure whether participants learned to detect the within-neighborhood spatial predictors of bitter Oomplet probability, we calculated a Spearman rank-order correlation coefficient $\rho_{S}$ between the order that a participant selected a house in a neighborhood rank_select_ and that house’s rank order of bitter Oomplet probability rank_house_. We refer to this as the *house bias (hb)* score, calculated as:


$$\begin{array}{c} hb=\rho_{S}({rank}_{\text{select}},{rank}_{\text{house}}) \end{array}$$ (4)

The magnitude of this value indicated how closely a participant followed the optimal exploration pattern inside a neighborhood. *hb* is highest in cases where the highest probability house patch that is currently unexplored and unharvested is always prioritized.

Learning effects analyses across rounds and associations between measures were performed using linear mixed-effects regression models. Participants were used as the random effects term.

In order to measure how well patch exit times conformed to the optimality defined by the MVT, we calculated local (patch) and global (expected) reward rates to derive a running *marginal reward rate*. The patch reward rate ($RR_{p}$) reflects the immediate rate of return within a given patch, defined as the total reward harvested in the current patch divided by the time spent in that patch.


$$\begin{array}{c} RR_{p}=\frac{\sum R_{patch}}{t_{patch}} \end{array}$$ (5)

The expected reward rate ($RR_{e}$) represents the baseline yield across a participant’s entire foraging experience, calculated as the cumulative reward collected across all patches and rounds divided by the time elapsed since the beginning of the first round.


$$\begin{array}{c} RR_{e}=\frac{\sum R_{T}}{t_{T}} \end{array}$$ (6)

Finally, the marginal reward rate quantifies the relative value of the current patch versus the overall rate of return in the environment. This is calculated as the difference between the patch and expected reward rates.


$$\begin{array}{c} \text{marginal reward rate}=RR_{p}-RR_{e} \end{array}$$ (7)

All code used to process and analyze data for this manuscript is available on GitHub (https://github.com/CoAxLab/BitterBusterTIER). All of the code and assets used to create and build the BitterBuster game are permanently archived on KiltHub (https://doi.org/10.1184/R1/33550681), and available on GitHub (https://github.com/CoAxLab/BitterBusterForagingPaperRelease).

## Supporting information

S1 Fig Exploration goal. Pre-round instruction screen presenting the objectives for the exploration task.
(TIF)

S2 Fig Selection goal. Pre-round instruction screen presenting the objectives for the selection task.
(TIF)

S3 Fig Houses. Pre-round instruction screen showing visual representations of houses (harvest points) within the virtual environment.
(TIF)

S4 Fig Neighborhoods. Pre-round instruction screen depicting a neighborhood (patch) using the in-game compass.
(TIF)

S5 Fig Scoring. Pre-round instruction screen summarizing the score-tracking user interface elements.
(TIF)

S6 Fig Controls. Pre-round instruction screen outlining the mouse and keyboard controls.
(TIF)
